# Updates in TECTA Mutation Spectrum: A Scoping Review

**DOI:** 10.3390/audiolres16050140

**Published:** 2026-09-21

**Authors:** Marianna Manuelli, Andrea Migliorelli, Stefano Pelucchi, Francesco Stomeo, Daniele Monzani, Silvia Palma, Giulia Vitetta, Chiara Bianchini, Andrea Ciorba

**Affiliations:** 1 ENT and Audiology Department, University Hospital of Ferrara, Via Aldo Moro 8, Cona, 44124 Ferrara, Italy; marianna.manuelli@ospfe.it (M.M.); mglndr1@unife.it (A.M.); stefano.pelucchi@unife.it (S.P.); francesco.stomeo@unife.it (F.S.); chiara.bianchini@unife.it (C.B.);; 2 ENT, Department of Surgical Sciences, Dentistry, Gynaecology and Paediatrics, University of Verona, Borgo Roma Hospital, 37134 Verona, Italy; 3 Audiology, Primary Care Department, AUSL of Modena, 41100 Modena, Italy; 4 Medical Genetics Unit, University Hospital of Parma, Via Gramsci 14, 43126 Parma, Italy; giulia.vitetta@studio.unibo.it

**Keywords:** TECTA, α-tectorin, hearing loss

## Abstract

**Background/Objectives**: The TECTA gene variant is one of the most common causes of nonsyndromic congenital hearing loss affecting the mid-frequency range. Situated on chromosome 11q22–q24, the TECTA gene encodes α-tectorin, a non-collagenous protein of the tectorial membrane. Pathogenetic variants of TECTA are relatively rare, and numerous variants have been described, complicating the delineation of a clear and recognizable clinical phenotype. This scoping review aims to provide an up-to-date overview of the known TECTA variants causing autosomal dominant sensorineural hearing loss (ADSNHL) and their associated audiological phenotypes. **Methods**: A review of the English-language literature on hearing loss associated with TECTA variants was conducted using PubMed, Scopus, Google Scholar, and MEDLINE databases, in accordance with the “Preferred Reporting Items for Systematic Reviews and Meta-Analysis (PRISMA)” criteria for scoping review. **Results**: Eighteen studies met all inclusion criteria. Overall, 44 unique TECTA variants were identified after recurrent reports of the same cDNA-level variant were consolidated. Of these, 14 were classified as pathogenic or likely pathogenic (P/LP), 9 as variants of uncertain significance (VUS), while the remaining 21 had conflicting, benign/likely benign, or unavailable classification. Domain-based analysis showed a marked prevalence of variants in the ZA domain (21 variants, 47.73%), followed by the ZP domain (14 variants, 31.82%) and the ENT domain (5 variants, 11.36%). Thirty-two variant occurrences were reported in association with hearing loss in the mid frequencies and 12 in the high frequencies. Stable hearing loss was reported for 15 variant occurrences, whereas progressive hearing loss was reported for 13. **Conclusions**: The identification and characterization of novel genetic variants associated with hearing loss are essential for improving phenotype–genotype correlations. Early identification of the genetic etiology of hearing loss is becoming increasingly important, particularly in light of recent advances in gene therapy for pediatric hearing loss.

## 1. Introduction

Among sensory disorders in humans, congenital bilateral sensorineural hearing loss (bilateral hearing loss ≥ 26 dB HL) is estimated to occur in 1 in 1000 screened newborns [1,2,3]. Genetic causes account for approximately 50% of cases of congenital hearing loss. Among these, approximately 30% of hearing impairments are syndromic and associated with specific clinical features, whereas the remaining 70% are nonsyndromic. Auditory function is complex and requires the interaction between many proteins. Consequently, variants in the genes encoding these proteins can result in hearing loss of varying severity. The classification of genetic variants represents a major challenge due to the ever-increasing number of genes identified as being associated with hearing loss. Moreover, some genetic disorders can exhibit phenotypic and allelic heterogeneity. Among cases of nonsyndromic hearing loss, autosomal dominant inheritance accounts for approximately 15–20%, with hearing loss typically being postlingual and progressive [4].

Variants in the TECTA gene have been reported as a cause of autosomal dominant mid-frequency hearing loss, affecting frequencies that are crucial for speech perception and intelligibility [4,5,6]. Autosomal Dominant (AD) missense variants in the TECTA gene (DFNA12, also designated as DFNA8) have been reported in 2.9–4% of all cases of autosomal dominant nonsyndromic sensorineural hearing loss (ADNSHL) [3,7]. Autosomal recessive variants in TECTA (DFNB21) have also been described [8].

Situated on chromosome 11q22–q24, the TECTA gene codes α-tectorin, a glycoprotein of the tectorial membrane (TM) [3,8]. α-tectorin is cleaved after translation to produce three covalently linked domains: the entactin domain (ENT); the large zonadhesin region (ZA); and the C-terminal zona pellucida (ZP) domain [7,9]. The TM mediates the deflection of the stereocilia of the sensory hair cells and the gating of mechanotransduction channels [9]. Variants in the TECTA gene can alter the mechanical properties of the TM, thereby impairing sound propagation and amplification [3]. Numerous TECTA variants have been described, complicating the delineation of a clear and recognizable clinical phenotype. In general, despite the identification of a large number of variants through next-generation sequencing (NGS), determining which variants are causally associated with hearing loss remains challenging. Therefore, detailed analysis of the audiological phenotypes of affected individuals is important for establishing genotype-phenotype correlations.

This scoping review aims to provide an up-to-date overview of known TECTA variants associated with ADSNHL and their genotype–audiological phenotype correlations, thereby helping clinicians promptly recognize and diagnose the condition.

## 2. Materials and Methods

This study was conducted in accordance with the Preferred Reporting Items for Systematic Reviews and Meta-Analyses extension for Scoping Reviews (PRISMA-ScR) guidelines [10,11]. The protocol for this review has been registered on the Open Science Framework (OSF) Registries with the Registration: URL accessed on 12 November 2025 https://osf.io/37dsr. PubMed, Scopus, Google Scholar, and MEDLINE databases were searched for relevant publications. The keywords “TECTA” or “alpha-tectorin” and “deafness” or “hearing loss” or “sensorineural hearing loss” were used. No time range was specified in the search; all articles published up to April 2026 were considered.

The inclusion criteria were (i) presence of a diagnosed TECTA variant, (ii) accurate description of the exact coding of the identified variant, (iii) characteristics of hearing loss associated with the variant, (iv) articles published in the English language.

The exclusion criteria were (i) studies in which hearing characteristics could not be determined from the information provided in the text, (ii) studies reporting a clear diagnosis of a different disease causing sensorineural hearing loss, (iii) reviews, (iv) studies describing autosomal recessive TECTA-associated hearing loss, (v) full texts not available(vi) full texts not in English.

Following study selection, the data were independently extracted by two authors (A.M. and M.M.) and reviewed by another author (A.C.). For each included article, the following information was collected: author/year, variant, protein change and current variant classification, exon and domain, reported characteristics of hearing loss, such as entity, laterality, stability/progression, and the eventual presence of other audiological features.

The audiological data to be extracted were selected according to the aims of the study. All data were collected in a Microsoft Excel^®^ (2024) database using a standardized data-charting form, which was pilot-tested on three randomly selected included articles. All team members contributed to the development of the data extraction form.

Titles and abstracts were screened first, followed by a full-text assessment of potentially eligible articles. Any disagreements were resolved through discussion and consensus, based on the relevance of the articles to the study aims and the availability of the required data. A predefined protocol was established to resolve any discrepancies arising during the selection process.

Hearing loss was classified by severity as mild (21–40 dB HL); moderate (41–70 dB HL); severe (71–90 dB HL); and profound (>90 dB HL), based on British Society of Audiology criteria [12]. Type of HL was classified based on the following: mid frequency = 0.5–2 kHz, and high frequency = 4–8 kHz. Concerning the features of hearing loss, progression or stability has also been reported, as indicated by the authors of the included studies.

All variants reported in the included studies were standardized according to the TECTA reference transcript NM_005422.4 [13]. We distinguished between reported variant occurrences and unique variants. A variant occurrence was defined as a specific nucleotide variant reported in a given publication, whereas a unique variant represented the same cDNA-level HGVS change counted only once across all publications. Recurrent variants were therefore identified on the basis of an identical cDNA-level nucleotide change according to NM_005422.4, rather than on the basis of an identical protein change alone. When the same variant was reported in more than one publication or family, it was counted only once in the analysis of the molecular variant spectrum. When the same variant was reported in multiple publications or families with differing classifications, the individual published classifications were retained for transparency and were not combined into a consensus classification.

For each unique variant, the classification reported in the original publication was recorded separately from the current ClinVar classification.

ClinVar was reviewed on 24 August 2026, recording the Variation ID, relevant RCV accession(s), classification, review status/star rating, and available submission information. When multiple classifications were available, the condition-specific classification relevant to autosomal dominant TECTA-associated hearing loss was prioritized. Conflicting interpretations were reported explicitly rather than collapsed into a single category. Variants classified as VUS, benign/likely benign, or with insufficient evidence were not included in the P/LP group. Absence of a ClinVar record was not considered equivalent to VUS; such variants were reported as “not reported in ClinVar”. Conversely, variants without a current ClinVar classification were included in the P/LP group only when the original publication provided sufficient evidence to support pathogenicity or likely pathogenicity, including clinical segregation and/or functional evidence, where available. Absence of a ClinVar record alone was not considered evidence of pathogenicity. Variants were summarized in two different groups: (pathogenic and likely pathogenic variants) and (uncertain, conflicting, benign/likely benign, or unavailable classification). Domain annotations were based on the original publication using NM_005422.4 and were considered descriptive rather than functional. For variants at domain boundaries, the original authors’ annotation was retained. For splice-site variants, domain assignment was considered not applicable unless explicitly linked to a specific domain in the original publication. The selection process is summarized in Figure 1.

## 3. Results

After the removal of duplicates and unrelated items, 22 articles were assessed for eligibility, of which four did not meet the inclusion criteria. Twenty--two studies were considered eligible for analysis; of these, 18 studies met the inclusion criteria and were included in the assessment. Most of the selected studies were published before 2015, and most papers (15) reported single cases or a small number of families. A total of 50 publication-specific TECTA variant occurrences were identified across the included studies and are summarized in Table 1. Recurrent variants reported in more than one publication were retained as separate publication-specific occurrences in this table. After recurrent reports of the same cDNA-level variant were consolidated, 44 unique TECTA variants were identified. The identified variants were distributed across the ENT, zonadhesin (ZA), and zona pellucida (ZP) domains of α-tectorin, with most reported variants located in the ZA and ZP regions.

Among the 44 unique TECTA variants identified in the included studies, 14 were classified as pathogenic or likely pathogenic (P/LP) and are summarized in Table 2, whereas 30 variants showed uncertain, conflicting, benign/likely benign, or unavailable classification and are reported in Table 3. Of the 14 P/LP variants, 12 were supported by current ClinVar classifications, while two were retained based on the clinical and/or functional evidence reported in the original literature, despite the absence of a ClinVar record. For variants with conflicting or condition-dependent ClinVar classifications, the interpretation relevant to autosomal dominant TECTA-associated hearing loss was considered. Variants without a ClinVar record were not considered equivalent to VUS and were reported as ‘not reported in ClinVar’. Please see also Supplementary Tables S1 and S2.

The variants were not evenly distributed across the gene but showed a clear concentration in specific exons (please see Table 4). Exons 19 and 11 were the most frequently represented in the dataset, with 5 unique variants each (11.36%).

Exon 7 followed in frequency, with 4 variants (9.09%), while exons 20, 21, and 10 contained 3 variants (6.82%). Exons 4, 8, 9, 14, and 17 each accounted for 2 variants (4.55%), whereas the remaining exons were represented by a single variant each (2.27% each).

Variants were most frequently located in the ZA domain (21 variants, 47.73%), followed by the ZP domain (14 variants, 31.82%) and the ENT domain (5 variants, 11.36%). One variant spanned the ZA/ZP domains (2.27%), while three splice-site variants could not be assigned to a specific domain (6.82%).

Most patients reported stable or slowly progressive sensorineural hearing loss in the mid frequencies (see also Table 1). Specifically, 32 variants were reported in association with hearing loss in the mid frequencies and 12 in the high frequencies. Stable hearing loss was reported for 15 variant occurrences, whereas progressive hearing loss was reported for 13. One patient carrying the c.5012C>T (p.Ser1671Leu) variant was reported to have profound unilateral hearing loss; however, this variant was classified as benign for autosomal dominant TECTA-associated hearing loss in ClinVar. Tinnitus was reported in 3 cases and vertigo in 5 cases.

Hearing loss was classified as stable or progressive based on changes observed during follow-up or according to the classification reported by the authors of the included studies. However, follow-up duration was not standardized across the studies; a minimum follow-up period of one year was required to classify hearing loss as stable.

## 4. Discussion

The TM is an extracellular matrix in the cochlea and plays a crucial role in hearing. Its mechanical connection with outer hair cells is essential to facilitate hair cell stimulation and sound transmission. Abnormalities of the TM have been associated with auditory disorders, with a prevalence ranging from 20 to 22% according to human temporal bone studies; both genetic etiologies and ototoxicity have been linked to TM abnormalities [8]. The TECTA gene, on chromosome 11q23.3, codes for α-tectorin, one of the main proteins involved in the organization of TM collagen fibrils.

While recessive variants of the TECTA gene result in a bite-shaped or U-shaped audiogram with very recognizable mid-frequency patterns, autosomal dominant variants of the TECTA gene can cause a range of hearing disorders [16]. Analysis of genotype-phenotype correlations indicates that the phenotypic presentation of such variants is influenced by the exact location of the involved domain. Variants affecting the ZP domain appear to be associated with prelingual, stable, mid-frequency hearing loss, whereas variants involving the zonadhesin (ZA) domain predominantly affect high frequencies [23,27]. However, these findings need to be confirmed by statistical analyses conducted on larger sample sizes.

In addition to the affected protein domain, the clinical phenotype appears to be influenced by variant type. Missense variants represent the most frequently reported class of TECTA variants in autosomal dominant forms. These variants cause single amino acid substitutions that may impair protein folding, disrupt intermolecular interactions, or alter the biomechanical properties of the TM. Their pathogenic effect is often mediated by a dominant-negative modality, as the mutant α-tectorin protein is incorporated into the extracellular matrix and interferes with normal tectorial membrane assembly. Clinically, missense variants are associated with moderate sensorineural hearing loss, involving either mid or high frequencies depending on the affected domain [7,14]. Missense variants involving cysteine residues have been associated with more severe or progressive phenotypes, particularly when located within the ZP domain, suggesting a stronger destabilizing effect on the protein architecture and extracellular matrix organization [27]. Splice-site variants represent another important variant class. These variants may alter normal mRNA processing, leading to exon skipping, intron retention, or the production of aberrant transcripts. The resulting phenotype appears more heterogeneous, reflecting the degree of residual normal transcript expression and the specific region affected. This may explain the variability in age of onset and progression observed in patients carrying splice-altering variants [14,18].

Truncating variants, including nonsense variants, frameshift variants, and large deletions, are more frequently associated with autosomal recessive inheritance. These variants usually result in loss-of-function through premature protein truncation or nonsense-mediated mRNA decay. Compared with dominant missense variants, truncating variants tend to produce earlier onset and more severe hearing impairment, often with congenital or prelingual presentation [7].

Since hearing loss associated with TECTA variants is usually not accompanied by other symptoms and may affect both mid-and high-frequency ranges, resulting in variable audiometric configurations rather than a single characteristic audiometric pattern, the diagnosis can be challenging, even for clinicians familiar with its clinical features. Mid- to high frequencies are essential for speech understanding; therefore, early recognition of these clinical features is relevant to promote language skills in children. On the other hand, a non-uniform definition of mid frequencies in the literature does not facilitate the identification of precise audiologic phenotypes.

Among the TECTA variants described by González-Aguado et al., one was associated with a clinical phenotype characterized by profound unilateral hearing loss. This finding represents an unusual phenotype compared with the moderate, symmetrical hearing-loss pattern more commonly reported in TECTA-associated autosomal dominant hearing loss. The analysis of a larger number of cases would help determine the frequency of this clinical feature [24]. Moreover, other predisposing factors in affected individuals may influence phenotypic expression.

An accurate and early diagnosis in children with a TECTA variant is of crucial importance, as hearing loss in childhood can lead to delays in speech and cognitive development.

Hearing loss associated with TECTA variants is generally moderate in severity; although this may be favorable in terms of hearing rehabilitation, it may also make the condition more difficult to recognize, potentially leading to delayed diagnosis. Indeed, moderate, stable hearing loss may remain undetected. Population-based studies have shown that 40–50% of sensorineural hearing loss is unilateral or mild bilateral [29,30]. Clinical features that may facilitate diagnosis include childhood onset of symmetrical hearing loss, which tends to remain stable over time and is associated with a down-sloping audiometric configuration [31]. Analysis of the data collected suggests that, in the case of asymmetric hearing loss or unilateral hearing loss, it is not possible to exclude the presence of a genetic disorder [32]. Genetic assessment is essential for an accurate diagnosis; however, it remains challenging, as current genetic testing approaches primarily focus on variants associated with high-frequency or syndromic hearing loss. Additionally, in the case of a mild phenotype, many families may not pursue genetic testing. However, TECTA should be considered in selected familiar/nonsyndromic childhood SNHL, particularly when the audiometric phenotype and inheritance pattern are suggestive, and should therefore be included in the genetic testing algorithm. In this regard, regional and ethnic differences in the frequency of the variants are important and must also be considered in the diagnostic assessment.

Drawbacks. The observations concerning genotype-phenotype correlations reported in this study are purely descriptive, based on the data collected. Various studies present a small number of cases and heterogeneous methodologies. Larger multicenter studies will be required to confirm these observations.

Another limitation of this study is the rarity of TECTA gene variants, which are more frequently reported in genetic research, often lacking in-depth audiological profiling. Furthermore, TECTA gene variants are not associated with syndromic clinical presentations or other systemic symptoms, and it is, therefore, challenging to establish precise recommendations for improving the diagnostic workup.

Future research should focus on improving our understanding of rare TECTA variants and developing a comprehensive and accurate database that could serve as a valuable resource for clinicians in the diagnosis and in the evaluation of complex cases. Accurate and precise genetic diagnosis and profiling is not only useful for diagnostic and research purposes, but can also be crucial in the era of gene therapy.

## 5. Conclusions

The identification and characterization of the genetic variants associated with hearing loss are essential for improving the diagnostic accuracy across different clinical conditions. Furthermore, in the era of gene therapy, the prompt identification of the genetic etiology of hearing loss is becoming increasingly important. Although rare, TECTA should be considered in selected familiar/nonsyndromic childhood SNHL, particularly when the audiometric phenotype and inheritance are suggestive. To date, TECTA variants are not associated with a typical audiological profile; however, the correlations between the affected domain and clinical presentation could suggest the possibility of formulating an increasingly accurate audiological phenotypic profile in the future.

## Figures and Tables

**Figure 1:**
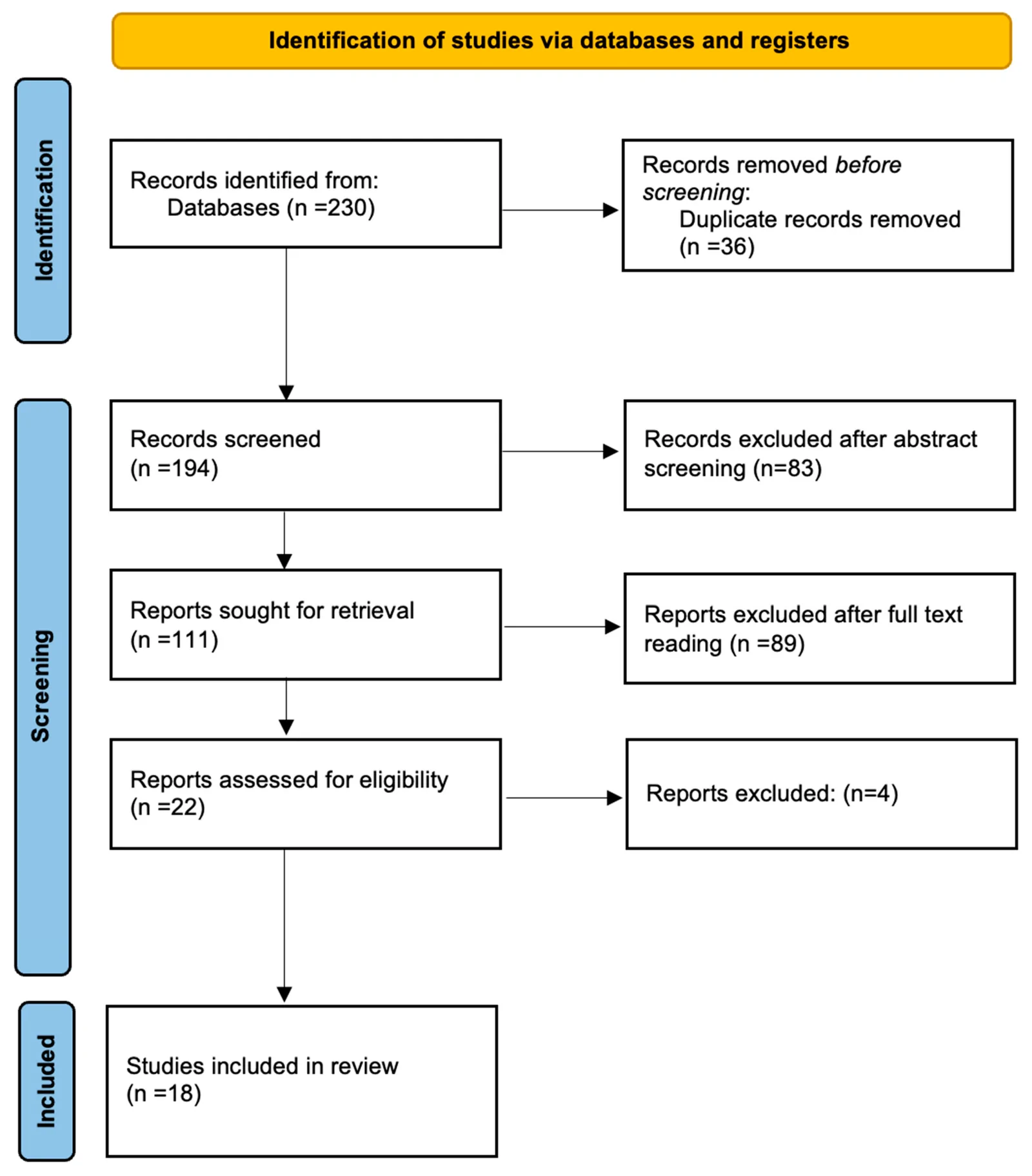
PRISMA flow diagram.

**Table 1:** TECTA variants reported in the included studies and associated audiological features. (NA = Data not available; HF = high frequencies, MF = mid frequencies).

Author/Year	TECTA Variant (NM_005422.4)	Protein Change	Exon	Domain	Hearing Loss	Other Audiological Features
Yamamoto [3] 2017	c.5668C>T	p.Arg1890Cys	19	ZP	MF, moderate	No
	c.1899C>A	p.Cys633Ter	9	ZA	MF, moderate	No
Yang [14] 2025	c.5999G>A	p.Gly2000Glu	20	ZP	Moderate-severe	No
Bai [1] 2014	c.6016G>T	p.Asp2006Tyr	21	ZP	Moderate-severe	No
Iwasaki [15] 2002	c.6062G>A	p.Arg2021His	21	ZP	Moderate, stable, MF	No
Nam [16] 2019	c.5668C>T	p.Arg1890Cys	19	ZP	Moderate, stable, MF	NA
Su [17] 2014	c.263_265del	p.Pro88del	4	ENT	Stable, HF	NA
Collin [18] 2008	c.5331G>A	p.Leu1777=	17	ZP	MF	NA
De Heer [19] 2009	c.5331G>A	p.Leu1777=	17	ZP	MF	Tinnitus in 3 cases; vertigo in 2 cases
Li [20] 2013	c.5945C>A	p.Ala1982Asp	20	ZP	MF, progressive	NA
Hildebrand [7] 2011	c.589G>A	p.Asp197Asn	5	ENT	MF-Stable	NA
	c.632T>C	p.Phe211Ser	6	ENT	MF-Stable	NA
	c.1084A>T	p.Ser362Cys	7	ZA	MF	NA
	c.1124delT	p.Val375Alafs*4	7	ZA	MF	NA
	c.1395T>G	p.Asn465Lys	8	ZA	MF-Progressive	NA
	c.1685C>T	p.Thr562Met	7	ZA	MF	NA
	c.2444C>T	p.Thr815Met	10	ZA	MF	NA
	c.2657A>G	p.Asn886Ser	10	ZA	HF-Progressive	NA
	c.3107G>A	p.Cys1036Tyr	11	ZA	MF-stable	NA
	c.3293C>T	p.Ala1098Val	11	ZA	HF	NA
	c.3406G>C	p.Asp1136His	11	ZA	HF	NA
	c.3743C>T	p.Pro1248Leu	12	ZA	HF	NA
	c.4549T>C	p.Cys1517Arg	14	ZA	HF-Progressive	NA
	c.5372C>G	p.Pro1791Arg	17	ZA	MF	NA
	c.5383+2T>G	Splice-site variant	-	NA	MF-Stable	NA
	c.5383+5_5383+8del	Splice-site variant	-	NA	HF-Progressive	NA
	c.5509T>G	p.Cys1837Gly	18	ZP	MF-Progressive	NA
	c.5597C>T	p.Thr1866Met	19	ZP	MF-Progressive	NA
	c.5600A>G	p.His1867Arg	19	ZP	MF-Progressive	NA
	c.5668C>T	p.Arg1890Cys	19	ZP	MF-Stable	NA
	c.5692T>C	p.Cys1898Arg	19	ZP	MF	NA
	c.5839C>T	p.Arg1947Cys	20	ZP	MF	NA
	c.6026T>C	p.Ile2009Thr	21	ZP	HF-Stable	NA
Sansovic [21] 2023	c.6183G>T	p.Arg2061Ser	22	ZP	MF-stable	NA
Sagong [22] 2010	c.950T>A	p.Val317Glu	7	ZA	MF	NA
	c.5597C>T	p.Thr1866Met	19	ZP	MF-Progressive	NA
Plantinga [23] 2006	c.248C>T	p.Thr83Met	4	ENT	MF	No
	c.5668C>T	p.Arg1890Cys	19	ZP	MF	No
González-Aguado [24] 2025	c.3107G>A	p.Cys1036Tyr	11	ZA	MF-Progressive	vertigo in 3 cases
	c.5383+6T>A	Splice-site variant	-	NA	MF	
	c.2825G>A	p.Arg942His	10	ZA	HF	
	c.5012C>T	p.Ser1671Leu	16	ZA/ZP	Profound unilateral	
	c.4171G>A	p.Ala1391Thr	13	ZA	MF-Stable	
	c.1443G>A	p.Met481Ile	8	ENT	HF-stable	
	c.2012G>A	p.Cys671Tyr	9	ZA	MF	
	c.2945T>C	p.Leu982Pro	11	ZA	MF	
Balciuniene [25] 1999	c.3169T>A	p.Cys1057Ser	11	ZA	HF-Progressive	NA
Pfister [26] 2004	c.4525T>G	p.Cys1509Gly	14	ZA	HF-Progressive	NA
Alloisio [27] 1999	c.4856G>C	p.Cys1619Ser	15	ZA	HF-Progressive	NA
Verhoeven [28] 1998	c.5609A>G	p.Tyr1870Cys	19	ZP	MF-Stable	NA

**Table 2:** Pathogenic and likely pathogenic TECTA variants.

Author	TECTA Variant (NM_005422.4)	Protein Change	ClinVar Classification	Review Status	Final Classification
Yamamoto [3]	c.5668C>T	p.Arg1890Cys	P/LP	2★	P/LP
Iwasaki [15]	c.6062G>A	p.Arg2021His	P/LP	2★	P/LP
Collin [18]	c.5331G>A	p.Leu1777=	LP	1★	LP
Li [20]	c.5945C>A	p.Ala1982Asp	LP	1★	LP
Hildebrand [7]	c.4549T>C	p.Cys1517Arg	LP	1★	LP
Hildebrand [7]	c.5383+5_5383+8del	Splice-site variant	P	2★	P
Hildebrand [7]	c.5509T>G	p.Cys1837Gly	P	0★	P
Hildebrand [7]	c.5597C>T	p.Thr1866Met	P/LP	2★	P/LP
González-A. [24]	c.3107G>A	p.Cys1036Tyr	LP (AD)	1★	LP
Balciuniene [25]	c.3169T>A	p.Cys1057Ser	P/LP	2★	P/LP
Alloisio [27]	c.4856G>C	p.Cys1619Ser	P	0★	P
Verhoeven [28]	c.5609A>G	p.Tyr1870Cys	P	1★	P
Sansovic [21]	c.6183G>T	p.Arg2061Ser	Not rep.	—	P/LP (literature)
González-A. [24]	c.5383+6T>A	Splice-site variant	Not rep.	—	P/LP (literature)

Note: ClinVar data were retrieved on 24 August 2026. For variants without a ClinVar record, pathogenic/likely pathogenic classification was based on the evidence reported in the original publication (★—rating scale according number of stars).

**Table 3:** TECTA variants with uncertain, conflicting, benign/likely benign, or unavailable classification.

Author	TECTA Variant (NM_005422.4)	Protein Change	Current Classification	Final Category
Yamamoto [3]	c.1899C>A	p.Cys633Ter	Not reported	NA/descriptive
Yang [14]	c.5999G>A	p.Gly2000Glu	Not reported	VUS/literature
Bai [1]	c.6016G>T	p.Asp2006Tyr	Not reported	NA/descriptive
Su [17]	c.263_265del	p.Pro88del	Not reported	NA/descriptive
Hildebrand [7]	c.589G>A	p.Asp197Asn	Not reported	VUS/literature
Hildebrand [7]	c.632T>C	p.Phe211Ser	Not reported	NA/descriptive
Hildebrand [7]	c.1084A>T	p.Ser362Cys	Not reported	VUS/literature
Hildebrand [7]	c.1124delT	p.Val375Alafs*4	Not reported	NA/descriptive
Hildebrand [7]	c.1395T>G	p.Asn465Lys	Not reported	NA/descriptive
Hildebrand [7]	c.1685C>T	p.Thr562Met	VUS vs. LB	Conflicting
Hildebrand [7]	c.2444C>T	p.Thr815Met	VUS for AD; VUS/LB overall	VUS/conflicting
Hildebrand [7]	c.2657A>G	p.Asn886Ser	VUS for AD; VUS/LB overall	VUS/conflicting
Hildebrand [7]	c.3293C>T	p.Ala1098Val	VUS	VUS
Hildebrand [7]	c.3406G>C	p.Asp1136His	VUS for AD; VUS/B overall	VUS/conflicting
Hildebrand [7]	c.3743C>T	p.Pro1248Leu	VUS	VUS
Hildebrand [7]	c.5372C>G	p.Pro1791Arg	VUS vs. LB	Conflicting
Hildebrand [7]	c.5383+2T>G	Splice-site variant	Not reported	NA/descriptive
Hildebrand [7]	c.5600A>G	p.His1867Arg	VUS	VUS
Hildebrand [7]	c.5692T>C	p.Cys1898Arg	LP vs. VUS	Conflicting
Hildebrand [7]	c.5839C>T	p.Arg1947Cys	P vs. VUS	Conflicting
Hildebrand [7]	c.6026T>C	p.Ile2009Thr	LP for AR DFNB21	AD/AR discordance
Sagong [22]	c.950T>A	p.Val317Glu	Not reported	NA/literature
Plantinga [23]	c.248C>T	p.Thr83Met	VUS for AD	VUS/condition-specific
González-Aguado [24]	c.2825G>A	p.Arg942His	VUS vs. LB	Conflicting
González-Aguado [24]	c.5012C>T	p.Ser1671Leu	Benign for AD	Benign
González-Aguado [24]	c.4171G>A	p.Ala1391Thr	Not reported	NA/descriptive
González-Aguado [24]	c.1443G>A	p.Met481Ile	VUS	VUS
González-A. [24]	c.2012G>A	p.Cys671Tyr	VUS	VUS
González-A. [24]	c.2945T>C	p.Leu982Pro	VUS for AD; VUS/LB overall	VUS/conflicting
Pfister [26]	c.4525T>G	p.Cys1509Gly	Not reported	NA/literature

Note: ClinVar data were retrieved on 24 August 2026. RCV/Variation IDs, submission details, and detailed conflict information are provided in the Supplementary ClinVar table.

**Table 4:** Distribution of variants by exons.

Exon Number	Number of Unique Variants	Percentage
19	5	11.36%
11	5	11.36%
7	4	9.09%
20	3	6.82%
21	3	6.82%
10	3	6.82%
4	2	4.55%
8	2	4.55%
9	2	4.55%
14	2	4.55%
17	2	4.55%
5	1	2.27%
6	1	2.27%
12	1	2.27%
13	1	2.27%
15	1	2.27%
16	1	2.27%
18	1	2.27%
22	1	2.27%
Non-exonic/splice-site	3	6.82%

## Data Availability

No new data were created or analyzed in this study. Data sharing is not applicable to this article.

## Supplementary Materials

The following supporting information can be downloaded at https://www.mdpi.com/article/10.3390/audiolres16050140/s1: Supplementary File S1: PRISMA ScR Checklist. Table S1: Detailed ClinVar assessment of pathogenic and likely pathogenic TECTA variants. Table S2: Detailed ClinVar assessment of uncertain, conflicting, benign/likely benign, or unavailable TECTA variants.
