# Supplementary material for: Updates in TECTA Mutation Spectrum: A Scoping Review
Source: Audiol Res. 2026 Sep 21;16(5):140. doi: 10.3390/audiolres16050140 (PMC13701212; doi:10.3390/audiolres16050140)
Supplement: Supplementary file 1 [file audiolres-16-00140-s001.zip › audiolres-4484577-supplementary.pdf]

Supplementary Table S1. Detailed ClinVar assessment of pathogenic and likely pathogenic TECTA variants

Author	cDNA	Protein	Original paper classification	ClinVar classification	Variation ID	RCV accession(s)	Review status	Conflict	Final classification
Yamamoto [3]; Nam [16]; Plantinga [23]	c.5668C>T	p.Arg1890Cys	LP/P	P/LP	7022	RCV000007437	2★; multiple submitters, no conflicts	No	P/LP
Iwasaki [15]	c.6062G>A	p.Arg2021His	LP/P	P/LP	7021	RCV000007436; RCV004017230	2★; multiple submitters, no conflicts	No	P/LP
Collin [18]; De Heer [19]	c.5331G>A	p.Leu1777=	LP	LP	7024	RCV000007439; RCV005628784	1★; criteria provided	No	LP
Li [20]	c.5945C>A	p.Ala1982Asp	LP	LP	3342518	RCV004729278	1★; single submitter	No	LP
Hildebrand [7]	c.4549T>C	p.Cys1517Arg	LP	LP	2412831	RCV003108262	1★; single submitter	No	LP
Hildebrand [7]	c.5383+5_5383+8del	Splice-site variant	NA	P	1185582	RCV002568953	2★; multiple submitters, no conflicts	No	P
Hildebrand [7]	c.5509T>G	p.Cys1837Gly	P	P	7020	AD-specific RCV	0★; no assertion criteria provided	No	P
Hildebrand [7]; Sagong [22]	c.5597C>T	p.Thr1866Met	LP/P	P/LP	236058	RCV000479439	2★; multiple submitters, no conflicts	No	P/LP
Hildebrand [7]; González-Aguado [25]	c.3107G>A	p.Cys1036Tyr	VUS/LP/P	LP for AD	517663	RCV000681522; RCV001755997	1★; AD RCV single submitter	Condition-dependent	LP
Balciuniene [26]	c.3169T>A	p.Cys1057Ser	LP/P	P/LP	7016	RCV000007431; RCV005089201	2★; multiple submitters, no conflicts	No	P/LP
Alloisio [28]	c.4856G>C	p.Cys1619Ser	P	P	7017	AD-specific RCV	0★; no assertion criteria provided	No	P
Verhoeven [29]	c.5609A>G	p.Tyr1870Cys	P	P	7014	RCV000007429	1★; single submitter	No	P
Sansovic [21]	c.6183G>T	p.Arg2061Ser	NA	Not reported	—	—	—	No ClinVar record	P/LP – literature
González-Aguado [25]	c.5383+6T>A	Splice-site variant	NA	Not reported	—	—	—	No ClinVar record	P/LP – literature

ClinVar reviewed 24 August 2026. RCVs are variant–condition records; the ClinVar Variation ID refers to the aggregated variant. Condition-specific interpretations relevant to autosomal dominant TECTA-related hearing loss were prioritized.

Supplementary Table S2. Detailed ClinVar assessment of uncertain, conflicting, benign/likely benign, or unavailable TECTA variants

Author	cDNA	Protein	Original paper classification	ClinVar classification	Variation ID	RCV accession(s)	Review status	Conflict	Final classification
Yamamoto [3]	c.1899C>A	p.Cys633Ter	NA	Not reported	—	—	—	No ClinVar record	NA / descriptive
Yang [14]	c.5999G>A	p.Gly2000Glu	VUS	Not reported	—	—	—	No ClinVar record	VUS / literature
Bai [1]	c.6016G>T	p.Asp2006Tyr	NA	Not reported	—	—	—	No ClinVar record	NA / descriptive
Su [17]	c.263_265del	p.Pro88del	NA	Not reported	—	—	—	No ClinVar record	NA / descriptive
Hildebrand [7]	c.589G>A	p.Asp197Asn	VUS	Not reported	—	—	—	No ClinVar record	VUS / literature
Hildebrand [7]	c.632T>C	p.Phe211Ser	NA	Not reported	—	—	—	No ClinVar record	NA / descriptive
Hildebrand [7]	c.1084A>T	p.Ser362Cys	VUS	Not reported	—	—	—	No ClinVar record	VUS / literature
Hildebrand [7]	c.1124delT	p.Val375Alafs*4	NA	Not reported	—	—	—	No ClinVar record	NA / descriptive
Hildebrand [7]	c.1395T>G	p.Asn465Lys	NA	Not reported	—	—	—	No ClinVar record	NA / descriptive
Hildebrand [7]	c.1685C>T	p.Thr562Met	LB/VUS	VUS (3) vs LB (1)	225485	RCV000490363; RCV000728512	1★; conflicting classifications	VUS vs LB	Conflicting
Hildebrand [7]	c.2444C>T	p.Thr815Met	LB/VUS	VUS for AD; VUS/LB overall	288521	RCV001336814; RCV000662344	1★; AD single submitter	VUS vs LB	VUS / conflicting
Hildebrand [7]	c.2657A>G	p.Asn886Ser	LB/VUS	VUS for AD; VUS/LB overall	229296	RCV001103540; RCV002500716; RCV000219728	1★; conflicting classifications	VUS vs LB	VUS / conflicting
Hildebrand [7]; González-Aguado [25]	c.3107G>A	p.Cys1036Tyr	VUS/LP/P	LP for AD	517663	RCV000681522; RCV001755997	1★; condition-dependent	Yes; AD-specific LP	Moved to 1A
Hildebrand [7]	c.3293C>T	p.Ala1098Val	VUS	VUS	1048876	RCV001354258	0★; no assertion criteria provided	No	VUS
Hildebrand [7]	c.3406G>C	p.Asp1136His	LB/VUS	VUS for AD; VUS/B overall	165360	RCV000763711; RCV001103637; RCV000726310	1★; conflicting classifications	VUS vs B	VUS / conflicting

Author	cDNA	Protein	Original paper classification	ClinVar classification	Variation ID	RCV accession(s)	Review status	Conflict	Final classification
Hildebrand [7]	c.3743C>T	p.Pro1248Leu	LB/VUS/LP	VUS	403527	RCV000454830	1★; single submitter	No	VUS
Hildebrand [7]	c.5372C>G	p.Pro1791Arg	LB/VUS	VUS (1) vs LB (1)	1178462	RCV001534750	1★; conflicting classifications	VUS vs LB	Conflicting
Hildebrand [7]	c.5383+2T>G	Splice-site variant	NA	Not reported	—	—	—	No ClinVar record	NA / descriptive
Hildebrand [7]	c.5600A>G	p.His1867Arg	VUS	VUS	2735779	RCV003557684	1★; single submitter	No	VUS
Hildebrand [7]	c.5692T>C	p.Cys1898Arg	VUS/LP	LP (1) vs VUS (1)	667414	RCV000826187; RCV002538245	1★; conflicting classifications	LP vs VUS	Conflicting
Hildebrand [7]	c.5839C>T	p.Arg1947Cys	VUS/P	P (1) vs VUS (3)	449555	RCV000520532; RCV000767069; RCV004748804	1★; conflicting classifications	P vs VUS	Conflicting
Hildebrand [7]	c.6026T>C	p.Ile2009Thr	AD reported	LP for AR DFNB21	3769085	RCV006700912	1★; single submitter	AD literature vs AR ClinVar	Condition/inheritance discordance
Sagong [22]	c.950T>A	p.Val317Glu	NA	Not reported	—	—	—	No ClinVar record	NA / literature
Plantinga [23]	c.248C>T	p.Thr83Met	VUS	VUS for AD; variant-level P/VUS/LB	813831	RCV001568737	1★; AD single submitter	Condition-dependent	VUS / condition-specific
González-Aguado [25]	c.2825G>A	p.Arg942His	LB/VUS	VUS (3) vs LB (1)	1446402	RCV001992827	1★; conflicting classifications	VUS vs LB	Conflicting
González-Aguado [25]	c.5012C>T	p.Ser1671Leu	B/LB/VUS	Benign for AD	45335	RCV000891765	1★; single submitter	Variant-level conflict across conditions	Benign
González-Aguado [25]	c.4171G>A	p.Ala1391Thr	NA	Not reported	—	—	—	No ClinVar record	NA / descriptive
González-Aguado [25]	c.1443G>A	p.Met481Ile	VUS	VUS	1214874	RCV001593349; RCV005749848	1★; single submitter	No	VUS
González-Aguado [25]	c.2012G>A	p.Cys671Tyr	VUS	VUS	1333705	RCV001808921	1★; single submitter	No	VUS
González-Aguado [25]	c.2945T>C	p.Leu982Pro	VUS	VUS for AD; VUS/LB overall	45324	RCV001852805	1★; AD single submitter	VUS vs LB at variant level	VUS / conflicting

Author	cDNA	Protein	Original paper classification	ClinVar classification	Variation ID	RCV accession(s)	Review status	Conflict	Final classification
Pfister [27]	c.4525T>G	p.Cys1509Gly	P / AD-associated	Not reported	—	—	—	No ClinVar record	NA / literature

ClinVar reviewed 24 August 2026. RCVs are variant–condition records; the ClinVar Variation ID refers to the aggregated variant. Condition-specific interpretations relevant to autosomal dominant TECTA-related hearing loss were prioritized.
